# Exploring the interplay between yeast cell membrane lipid adaptation and physiological response to acetic acid stress

**DOI:** 10.1128/aem.01212-24

**Published:** 2024-11-13

**Authors:** Fei Wu, Maurizio Bettiga, Lisbeth Olsson

**Affiliations:** 1 Department of Life Sciences, Division of Industrial Biotechnology, Chalmers University of Technology, Gothenburg, Sweden; 2 Italbiotec Srl Benefit Corporation, Innovation Unit, Milan, Italy; Washington University in St. Louis, St. Louis, Missouri, USA

**Keywords:** diacylglycerol, Dgkα, membrane permeability, energy burden, glucose uptake rate, lipidomics

## Abstract

Acetic acid is a byproduct of lignocellulose pretreatment and a potent inhibitor of yeast-based fermentation processes. A thicker yeast plasma membrane (PM) is expected to retard the passive diffusion of undissociated acetic acid into the cell. Molecular dynamic simulations suggest that membrane thickness can be increased by elongating glycerophospholipids (GPL) fatty acyl chains. Previously, we successfully engineered *Saccharomyces cerevisiae* to increase GPL fatty acyl chain length but failed to lower acetic acid net uptake. Here, we tested whether altering the relative abundance of diacylglycerol (DAG) might affect PM permeability to acetic acid in cells with longer GPL acyl chains (DAG*^EN^*). To this end, we expressed diacylglycerol kinase α (*DGKα*) in DAG*^EN^*. The resulting DAG*^EN^*_Dgkα strain exhibited restored DAG levels, grew in medium containing 13 g/L acetic acid, and accumulated less acetic acid. Acetic acid stress and energy burden were accompanied by increased glucose uptake in DAG*^EN^*_Dgkα cells. Compared to DAG*^EN^*, the relative abundance of several membrane lipids changed in DAG*^EN^*_Dgkα in response to acetic acid stress. We propose that the ability to increase the energy supply and alter membrane lipid composition could compensate for the negative effect of high net acetic acid uptake in DAG*^EN^*_Dgkα under stressful conditions.

**IMPORTANCE:**

In the present study, we successfully engineered a yeast strain that could grow under high acetic acid stress by regulating its diacylglycerol metabolism. We compared how the plasma membrane and total cell membranes responded to acetic acid by adjusting their lipid content. By combining physiological and lipidomics analyses in cells cultivated in the absence or presence of acetic acid, we found that the capacity of the membrane to adapt lipid composition together with sufficient energy supply influenced membrane properties in response to stress. We suggest that potentiating the intracellular energy system or enhancing lipid transport to destination membranes should be taken into account when designing membrane engineering strategies. The findings highlight new directions for future yeast cell factory engineering.

## INTRODUCTION

Microbial fermentation of plant biomass has attracted considerable attention due to the growing demand for renewable resources and increasing concerns about climate change (1, 2). However, byproducts such as weak acids generated during the pretreatment process are strong inhibitors of fermenting microorganisms and slow the bioconversion of cellulosic hydrolysates (3, 4). Acetic acid is a common weak acid and industrially relevant concentration affects xylose consumption in the fermentation (4, 5). Also, acetic acid accumulation triggers intracellular acidification and even apoptosis in yeast (6). Therefore, minimizing the intracellular concentration of acetic acid is expected to improve the efficiency of lignocellulosic fermentation. In yeast-based fermentation, pH is typically around 5, allowing undissociated acetic acid to enter the cell predominantly via passive diffusion across the plasma membrane (PM) (7). Membrane lipids, which include glycerophospholipids (GPL), sphingolipids (SPL), and sterols, determine the biophysical properties of membranes (8, 9). If it is enriched with SPL and sterols, the PM is thicker, more rigid, and tightly packed (9, 10), thereby slowing the passive diffusion process (11).

Comparative lipidomics between the highly acid-tolerant yeast *Zygosaccharomyces bailii* and the laboratory yeast *Saccharomyces cerevisiae* uncovered more SPL and longer acyl chains on GPL in *Z. bailii*, pointing to a correlation between SPL abundance, GPL acyl chain length, and tolerance to acetic acid (12). Molecular dynamic simulations indicated that enrichment with SPL or GPL containing very long-chain fatty acids could lead to a thicker and less permeable membrane (13, 14). All this evidence suggested that engineering thicker PMs by elongating GPL fatty acyl chains and/or increasing SPL abundance could decrease permeability to acetic acid.

In a previous study, *S. cerevisiae* CEN.PK 115D was engineered to express *Arabidopsis thaliana* fatty acid elongase 1 (AtFae1) and glycerol-3-phosphate acyltransferase 5 (AtGpat5) (14). The resulting DAG*^EN^* strain presented longer fatty acyl chains on GPL but that did not diminish the net uptake of acetic acid. In fact, DAG*^EN^* was unable to grow in medium containing 13 g/L acetic acid, which also coincided with an abnormally high intracellular level of diacylglycerol (DAG) (14). DAG is an important membrane component and a key regulator of lipid metabolism and lipid-mediated signaling. Molecular dynamic simulations and liposome analysis suggested that a higher DAG content could disrupt the cell membrane lamellar structure, by altering either the curvature or thickness of the membrane (15, 16). In yeast, the accumulation of DAG could trigger endomembrane disorders (17) or even cell death (18). Nevertheless, the correlation between the increased length of the lipid acyl chains and a thicker membrane makes DAG*^EN^* an ideal strain for further metabolic engineering. Here, we hypothesized that restoring DAG levels in DAG*^EN^* cells could deliver a yeast strain, whose thicker PM could limit acetic acid uptake.

**Fig 1:**
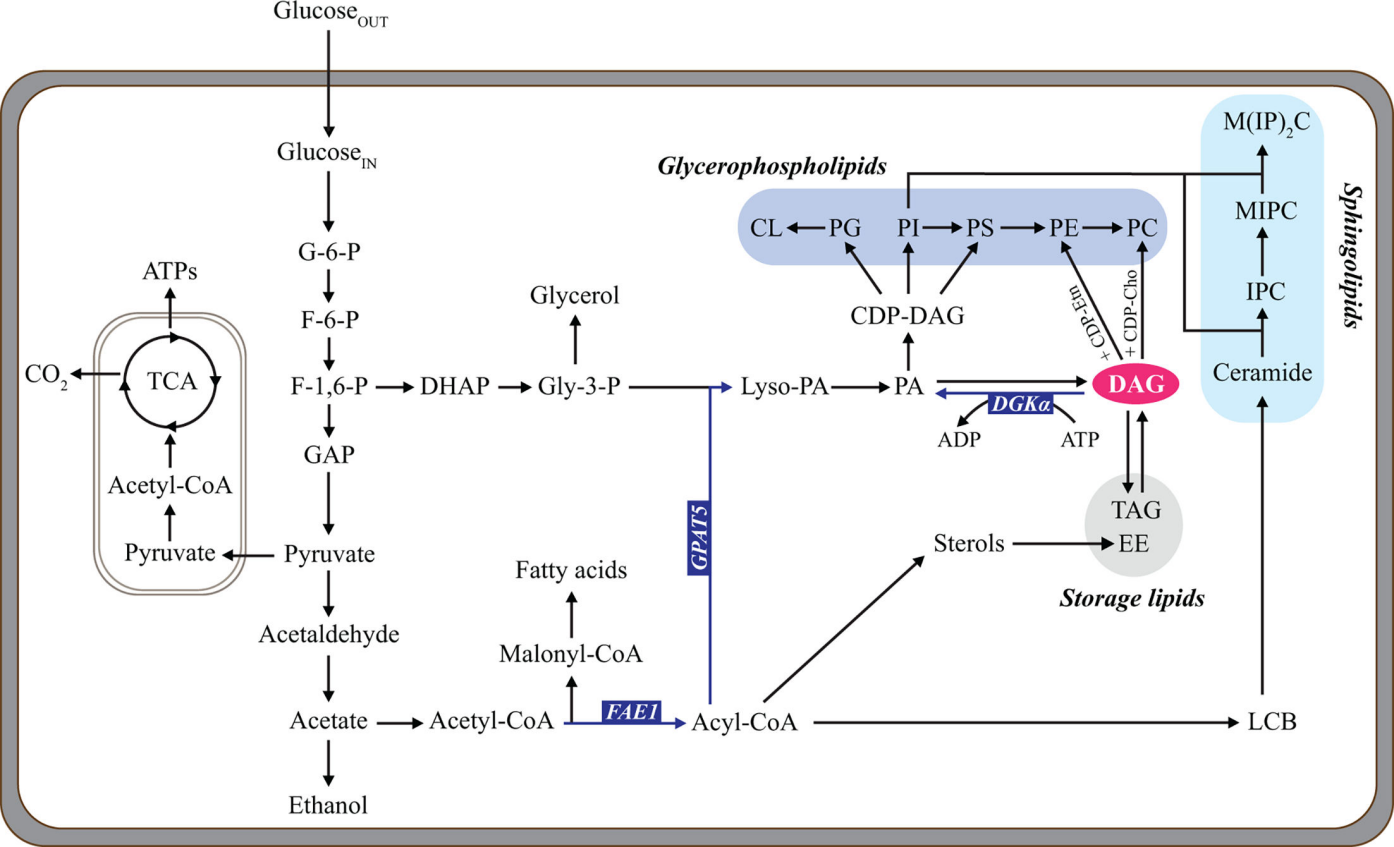
Illustration of membrane lipids *de novo* biosynthesis in DAG*^EN^*_Dgkα. Glycerophospholipids, sphingolipids, and storage lipids are shaded in blue, cyan, and gray, respectively. *DGKα*, diacylglycerol kinase α; *FAE1*, fatty acid elongase 1; GPAT5, glycerol-3-phosphate acyltransferase 5; DAG, diacylglycerol; PA, phosphatidic acid; Lyso-PA, lysophosphatidate; CDP-DAG, cytidine diphosphate diacylglycerol; PG, phosphatidylglycerol; CL, cardiolipin; PI, phosphatidylinositol; PS, phosphatidylserine; PE, phosphatidylethanolamine; PC, phosphatidylcholine; CDP-Etn, cytidine diphosphate ethanolamine; CDP-Cho, cytidine diphosphate choline; TAG, triacylglycerol; SE, ergosterol ester; Gly-3-P, glycerol 3-phosphate; LCBs, long-chain bases; IPC, inositol phosphorylceramide; MIPC, mannosyl-inositol phosphorylceramide; M(IP)_2_C, mannosyl-di-(inositol phosphoryl) ceramide; Acyl-CoA, acyl-coenzyme A; Acetyl-CoA, acetyl-coenzyme A; Malonyl-CoA, malonyl-coenzyme A; G-6-P, glucose 6-phosphate; F-6-P, fructose 6-phosphate; F-1,6-P, fructose 1,6-bisphosphate; GAP, glyceraldehyde 3-phosphate; TCA, citric acid cycle; DHAP, dihydroxyacetone phosphate; Gly-3-P, glycerol-3-phosphate.

In yeast, DAG could be either acylated to triacylglycerol (TAG) to form storage lipids or phosphorylated to phosphatidic acid (PA) for cell membrane lipid biosynthesis (19). The strain engineering strategy proposed in the present study aimed to convert DAG to PA, thereby tilting the balance towards membrane lipids. In yeast, the phosphorylation of DAG to PA is catalyzed by a CTP-dependent diacylglycerol kinase encoded by *DGK1* (20). Instead, in bacteria, plants, and animals, diacylglycerol kinase utilizes ATP as the phosphate donor (21). CTP is an essential precursor for the biosynthesis of membrane phospholipids from CDP-DAG, CDP-choline, and CDP-ethanolamine (22), but CTP requires ATP for its production (23). Here, *Escherichia coli* diacylglycerol kinase α (Dgkα) was selected and heterologously expressed in DAG*^EN^* to minimize a potential CTP consumption stress during DAG metabolism (Fig. 1).

In the present study, expression of Dgkα in DAG*^EN^* lowered the relative abundance of DAG and rescued the growth defect of DAG*^EN^* when grown in medium containing 13 g/L acetic acid. The resulting DAG*^EN^*_Dgkα strain accumulated less acetic acid even though its PM was more permeable than that of DAG*^EN^* and control cells. This could be attributed to more flexibility of DAG*^EN^*_Dgkα cells in adjusting their membrane lipid composition when faced with acetic acid stress. DAG*^EN^*_Dgkα cells showed an almost complete abolishment of growth on glycerol, a non-fermentable carbon source, suggesting affected mitochondrial activity. Given the increased glucose uptake rate in DAG*^EN^*_Dgkα in response to acetic acid stress, we believe that active membrane lipid adaptation and an appropriate energy supply are both essential to ensure physiological performance under stressful conditions.

## RESULTS

Three yeast strains were investigated in the present study: *S. cerevisiae* CEN.PK115D with empty vector control (CNTR), DAG-enriched DAG*^EN^*, and Dgkα-expressing DAG*^EN^*_Dgkα. Unless mentioned otherwise, two conditions were applied: (i) standard growth on minimal medium (pH 5.0) containing 20 g/L glucose, and (ii) acetic acid stress, whereby 9 g/L acetic acid was added to the above medium.

### The growth defect of DAG*^EN^* in medium containing 13g/L acetic acid is rescued by expressing *DGKα*

**Fig 2:**
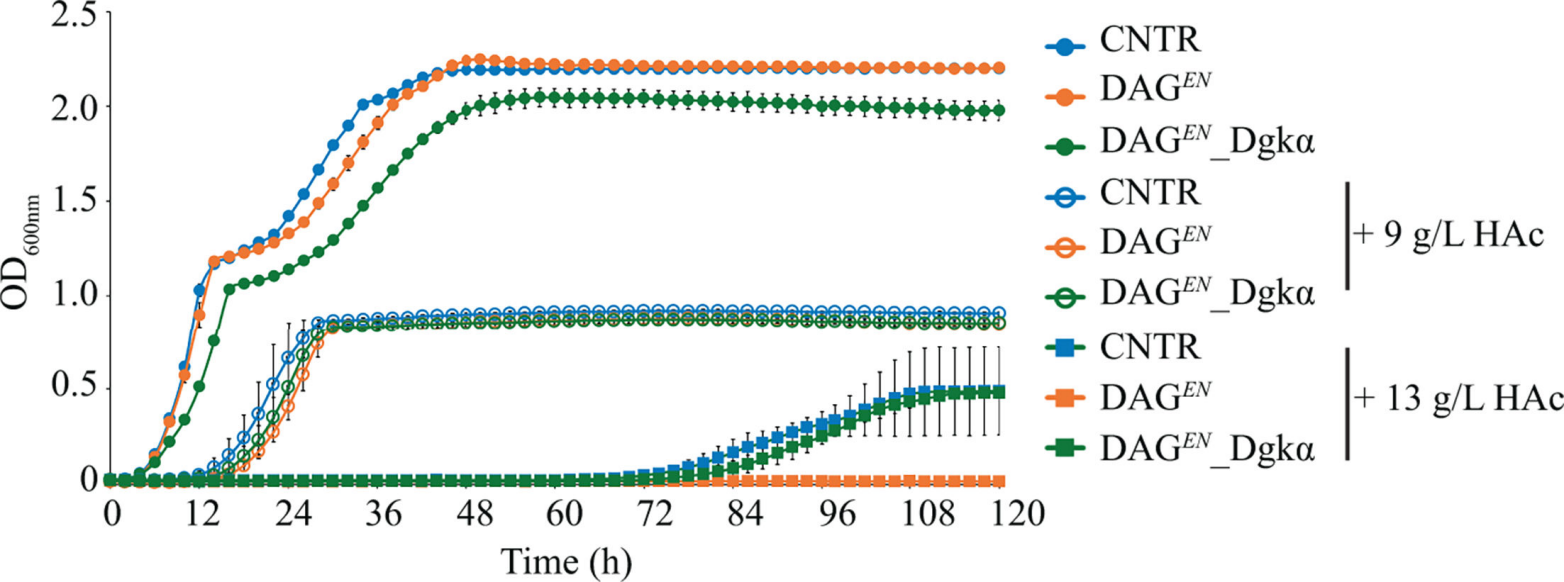
Expression of *DGKα* rescues the growth defect of DAG*^EN^* under 13 g/L acetic acid stress. Growth curve of CNTR, DAG*^EN^*, and DAG*^EN^*_Dgkα cells cultivated in minimal medium containing the indicated acetic acid (HAc) concentration. Cultivations were performed the high-throughput growth profiler. Data represent the means from three independent experiments and error bars the standard deviation.

Previously, relatively high levels of DAG in DAG*^EN^* cells were linked to poor growth in 13 g/L acetic acid-containing medium. To verify this hypothesis, we constructed strain DAG*^EN^*_Dgkα with the intention of lowering its DAG content and assessing growth under acetic acid stress. CNTR, DAG*^EN^*, and DAG*^EN^*_Dgkα were grown by high-throughput means in minimal medium containing different amounts of acetic acid (0 g/L, 9 g/L, or 13 g/L). In the absence of acetic acid, DAG*^EN^*_Dgkα cells reached the diauxic shift with a 4-h delay and attained the lowest final cell density (OD_600nm_ = 2.05 ± 0.05 for DAG*^EN^*_Dgkα, OD_600nm_ = 2.22 ± 0.01 for DAG*^EN^*, and OD_600nm_ = 2.20 ± 0.01 for CNTR) (Fig. 2). This effect might be due to metabolic burden, a feature often encountered in engineered strains (24). Following this assumption, some ATP was used to convert DAG to PA in DAG*^EN^*_Dgkα, thereby limiting the ATP available for cell growth. In the presence of 9 g/L acetic acid, no significant growth difference could be observed among the three strains (Fig. 2). Instead, with 13 g/L acetic acid, DAG*^EN^*_Dgkα and CNTR exhibited a similar 70-h lag phase, growth rate, and final cell density (Fig. 2), whereas DAG*^EN^* failed to grow at all (Fig. 2), confirming earlier reports (14). These data suggest that the growth defect of DAG*^EN^* in the presence of 13 g/L acetic acid could be rescued by expressing *DGKα*. Hence, DAG*^EN^*_Dgkα was selected for further investigation of its membrane properties.

### DAG*^EN^*_Dgkα achieves lower intracellular acetic acid accumulation

**Fig 3:**
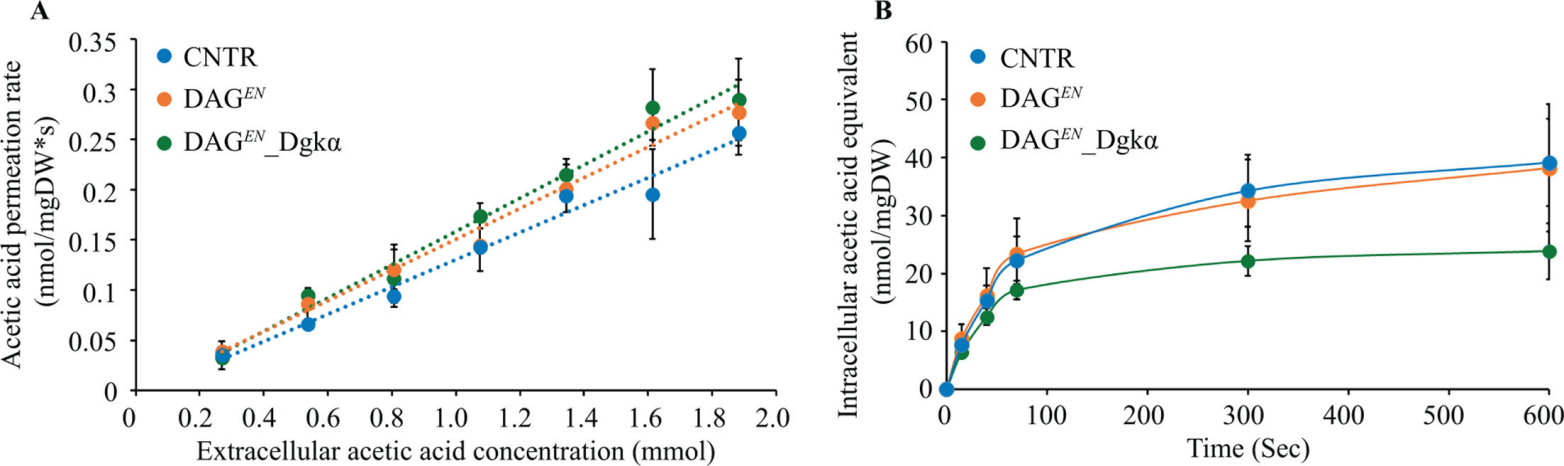
DAG*^EN^*_Dgkα shows the highest acetic acid diffusion rate but the lowest intracellular accumulation. (**A**) Acetic acid uptake kinetics of CNTR, DAG*^EN^*, and DAG*^EN^*_Dgkα in the presence of 0.2–2 mM extracellular acetic acid. (**B**) Acetic acid uptake with an initial extracellular acetic acid concentration of 2 mM (pH 5). The average sample response is given for each time point. Data represent the means from three independent experiments and error bars the standard deviation. Student’s *t*-test was applied for intergroup comparisons and no significant difference (*P* > 0.05) was observed. DW, cell dry weight.

The present engineering approach aimed to decrease PM permeability and, therefore, prevent the passive diffusion of undissociated acetic acid into cells (14). To measure acetic acid uptake, exponentially growing CNTR, DAG*^EN^*, and DAG*^EN^*_Dgkα cells were incubated in the presence of 0.2–2 mM [1-^14^C] acetic acid at pH 5.0 for 30 s. This short exposure time is used for determining the initial rate of acetic acid uptake before the effect of accumulated intracellular acetic acid becomes significant (14, 25). A linear correlation between the extracellular acetic acid concentration and its permeation rate was observed in all strains (Fig. 3A), suggesting that its uptake within this concentration range occurred predominantly as passive diffusion. The uptake rate constant was determined as the slope of the curve for the acetic acid permeation rate versus extracellular acetic acid concentration (Fig. 3A). Consistent with a previous study, whereby the slope was higher for DAG-enriched cells (0.15 ± 0.01) than CNTR cells (0.14 ± 0.01) (14), DAG*^EN^*_Dgkα exhibited an even higher slope (0.17 ± 0.02) and, therefore, net acetic acid uptake rate (Fig. 3A). Accordingly, expression of *DGKα* successfully rescued the growth defect of DAG*^EN^* cells in the presence of 13 g/L acetic acid, but without decreasing PM permeability to acetic acid.

Next, the intracellular acetic acid equivalent concentration as a function of time was determined by measuring the radioactive decay following the addition of 2 mM extracellular [1-^14^C] acetic acid. DAG*^EN^*_Dgkα, DAG*^EN^*, and CNTR cells reached a similar intracellular acetic acid equivalent number at 15 s, indicating a comparable acetic acid uptake at the initial stage. From 45 s until 10 min, DAG*^EN^*_Dgkα attained the lowest intracellular acetic acid concentration compared to DAG*^EN^*, and CNTR (Fig. 3B). This implied that DAG*^EN^*_Dgkα had either the lowest ability to accumulate acetate or the highest capacity to excrete it. Hence, the restored growth ability of DAG*^EN^*_Dgkα under 13 g/L acetic acid stress was likely due to lower intracellular acetic acid accumulation.

### DAG*^EN^*_Dgkα cells display impaired intracellular metabolic activity under standard growth conditions

**Fig 4:**
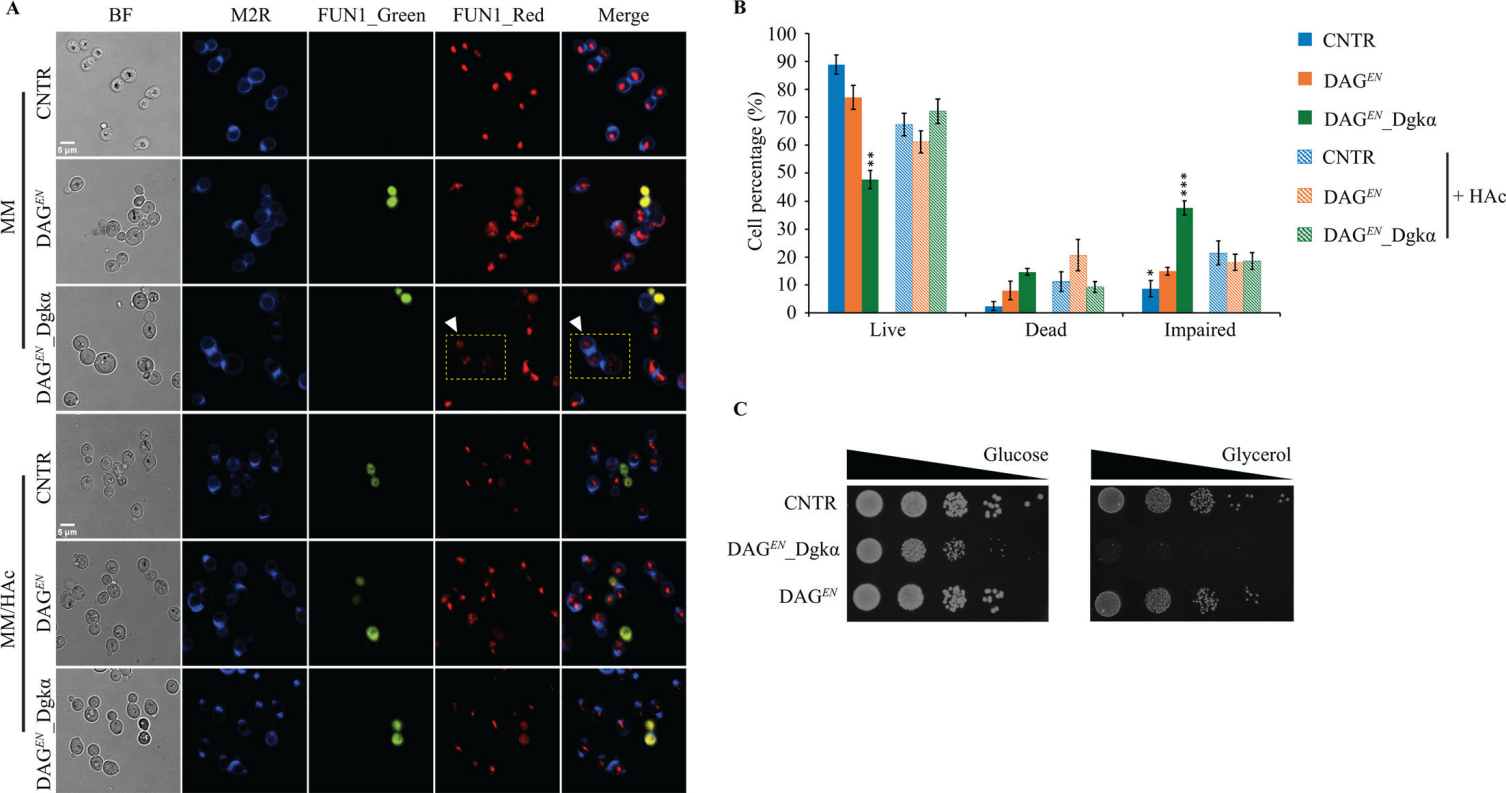
Expression of *DGKα* negatively affects intracellular metabolic activity and leads to a growth defect on glycerol. (**A**) Confocal laser scanning fluorescence microscopy images of the indicated strains grown on minimal medium (MM) supplemented or not with 9 g/L acetic acid (HAc). Yellow squares enclose impaired cells. (**B**) Percentage of live, dead, or impaired cells under the two conditions. Data represent the mean ± standard deviation (*n* = 3, with 300 cells per experiment). Student’s *t-test* was applied for intergroup comparisons. *P < 0.05, **P < 0.01, and ***P < 0.001. Scale bars: 5 µm. (**C**) Spot assay for strains grown on YP plates supplemented with either glucose or glycerol (pH 5.0) at 30°C for 4 days.

The high acetic acid uptake rate but low intracellular acetic acid accumulation in DAG*^EN^*_Dgkα led us to investigate membrane integrity in this strain. CNTR, DAG*^EN^*, and DAG*^EN^*_Dgkα cells growing exponentially in either standard or acid conditions were stained with the Live/Dead Yeast Viability Kit and checked by confocal laser scanning microscopy to assess cell viability, an indirect indicator of cell membrane integrity (26). The fluorescent Calcofluor White M2R stain revealed intact cell walls in all tested strains under both growth conditions (Fig. 4A). The two-color fluorescent dye FUN-1 formed red cylindrical intravacuolar structures (CIVS) via an ATP-dependent process (27) in metabolically active cells (FUN 1_Red panel, Fig. 4A) or remained cytosolic and green in dead cells (FUN 1_Green panel, Fig. 4A). Red punctate structures were observed in cells (FUN 1_Red panel, Fig. 4A), which were deemed still alive but with impaired vacuolar protein sorting (28).

Under standard conditions, DAG*^EN^*_Dgkα exhibited the lowest percentage of live cells (47.8% ± 3.2% in DAG*^EN^*_Dgkα, 88.9% ± 3.4% in CNTR, and 77.1% ± 4.3% in DAG*^EN^*), but the highest percentage of dead (14.7% ± 1.2% in DAG*^EN^*_Dgkα, 2.4% ± 1.6% in CNTR, and 8% ± 3.3% in DAG*^EN^*) and impaired (37.6% ± 2.5% in DAG*^EN^*_Dgkα, 8.7% ± 2.8% in CNTR, and 14.9% ± 1.4% in DAG*^EN^*) cells compared with CNTR and DAG*^EN^* (Fig. 4B). Such high proportion of dead cells suggested that genetic engineering had affected metabolic activity, depleting cells of ATP for CIVS formation. At the same time, the abundance of impaired DAG*^EN^*_Dgkα cells (Fig. 4A and B) was possibly related to a defect in vacuolar protein sorting (28), which might result from affected vacuolar membrane properties caused by altered membrane lipid metabolism. By contrast, under 9 g/L acetic acid stress, all three strains displayed comparable numbers of live, dead, and impaired cells (Fig. 4B), indicating similar intracellular metabolic activity.

Mitochondria are responsible for producing ATP via the citric acid (TCA) cycle (Fig. 1). *S. cerevisiae* is unable to utilize glycerol, a non-fermentable compound, as a sole carbon source when its mitochondria are not fully functional (29, 30). To determine whether the energy burden in DAG*^EN^*_Dgkα was related to mitochondrial activity, CNTR, DAG*^EN^*, and DAG*^EN^*_Dgkα were spotted on YP plates with either glucose or glycerol as the carbon source. After 4 days of incubation, DAG*^EN^*_Dgkα exhibited a slight growth defect on glucose, but lost entirely the ability to grow on glycerol (Fig. 4C). The spot assay data suggested that Dgkα-involved membrane lipid metabolism regulation might negatively affect mitochondrial function, which further cause an energy burden in DAG*^EN^*_Dgkα cells under standard growth conditions.

### The glucose uptake rate in DAG*^EN^*_Dgkα cells increases in response to acetic acid

To further characterize the physiological response of CNTR, DAG*^EN^*, and DAG*^EN^*_Dgkα during growth in the presence of acetic acid, all three strains were cultivated in triplicates in bioreactors under either standard or acetic acid conditions. The maximum specific growth rate (μ_Max_), biomass, and extracellular metabolite levels were measured and calculated.

As revealed by fermentation profiles (Fig. S1), CNTR, DAG*^EN^*, and DAG*^EN^*_Dgkα exhibited similar glucose consumption and byproduct formation under both standard and acetic acid conditions. A 6-h delay in consuming glucose was observed in all three strains when cultivated with 9 g/L acetic acid (Fig. S1), which might be a result of adaptation to acid stress. In the absence of acetic acid, DAG*^EN^*_Dgkα achieved the lowest µ_Max_ (0.29 ± 0.05 h^−1^) compared to CNTR (0.43 ± 0.03 h^−1^) and DAG*^EN^* (0.37 ± 0.03 h^−1^) (Table 1). By contrast, under acetic acid stress, the µ_Max_ increased by 10% in DAG*^EN^*_Dgkα cells (0.32 ± 0.03 h^−1^), while decreasing by 30% in CNTR (0.30 ± 0.02 h^−1^) and 16% in DAG*^EN^* (0.31 ± 0.02 h^−1^) cells (Table 1).

**TABLE 1:** Physiological data obtained from aerobic batch fermentations^*a*^^,^^*b*^

		CNTR		DAG*^EN^*		DAG*^EN^*_Dgkα	
		MM	MM/HAc	MM	MM/HAc	MM	MM/HAc
µ_Max_	h^−1^	0.43 ± 0.03	0.30 ± 0.02**	0.37 ± 0.03	0.31 ± 0.02	0.29 ± 0.05	0.32 ± 0.03
q_Glucose_	cmol × cmol DW^−1^ × h^−1^	2.77 ± 0.36	2.71 ± 0.15	2.43 ± 0.34	2.85 ± 0.31	2.14 ± 0.16	3.01 ± 0.29*
Y_X/S_	cmol × cmol^−1^	0.16 ± 0.01	0.11 ± 0.01**	0.15 ± 0.01	0.11 ± 0.01*	0.14 ± 0.01	0.11 ± 0.00*
Y_Glycerol/S_	cmol × cmol^−1^	0.03 ± 0.00	0.01 ± 0.00**	0.03 ± 0.00	0.01 ± 0.00**	0.05 ± 0.00	0.02 ± 0.00***
Y_EtOH/S_	cmol × cmol^−1^	0.49 ± 0.03	0.53 ± 0.02	0.51 ± 0.05	0.55 ± 0.01	0.53 ± 0.04	0.56 ± 0.02
Y_Pyruvate/S_	cmol × cmol^−1^	0.004 ± 0.00	0.005 ± 0.00	0.003 ± 0.00	0.004 ± 0.00**	0.003 ± 0.00	0.004 ± 0.00
Y_CO2/S_	cmol × cmol^−1^	0.37 ± 0.06	0.42 ± 0.04	0.35 ± 0.02	0.41 ± 0.04	0.36 ± 0.03	0.39 ± 0.02
Y_Acetate/S_	cmol × cmol^−1^	0.02 ± 0,00	N/A	0.02 ± 0.00	N/A	0.02 ± 0.00	N/A

^*a*^ CNTR, DAG*^EN^*, and DAG*^EN^*_Dgkα were cultured in minimal medium (MM) containing 0 g/L or 9 g/L acetic acid (HAc) using bioreactors. The pH was maintained at 5.

^*b*^ Data represent the means ± standard deviation (*n* = 3). Student’s *t-test* was applied when comparing MM and MM/HAc for each strain. *P < 0.05, **P < 0.01, and ***P < 0.001.

All three strains displayed significantly lower biomass and glycerol yields in response to acetic acid stress (Table 1), in line with previous studies (12, 31). Under standard conditions, DAG*^EN^*_Dgkα cells attained a lower biomass yield (Y_X/S_) than DAG*^EN^* and CNTR. The Y_X/S_ dropped similarly for all strains when they were cultivated with 9 g/L acetic acid (Table 1). Compared with CNTR and DAG*^EN^*, DAG*^EN^*_Dgkα exhibited the highest glycerol yield with or without acetic acid (Table 1). This result might be an indirect effect of Dgkα, whose conversion of DAG to PA diverts the carbon flux towards the glycerol biosynthesis precursor glycerol-3-phosphate (Fig. 1). Notably, in response to acetic acid, DAG*^EN^*_Dgkα augmented significantly its glucose uptake rate. We hypothesized that the higher glucose demand could be explained by a lower ATP yield caused by inefficient mitochondrial function and/or by increased ATP consumption due to active acetate and proton extrusion.

CNTR, DAG*^EN^*, and DAG*^EN^*_Dgkα showed similar patterns of acetate metabolism, in which cells started to produce acetate after 6 h of cultivation and used up all the acetate within 24 h under standard growth conditions (Fig. S1A, C, E). No obvious consumption of acetic acid was observed in any of the strains when cultivated in medium containing 9 g/L acetic acid (Fig. S1B, D, F). In addition, the yields of ethanol, pyruvate, and CO_2_ were not affected in CNTR, DAG*^EN^*, and DAG*^EN^*_Dgkα under either growth condition (Table 1). These data indicated that an energy shortage was the main source of stress for DAG*^EN^*_Dgkα and could be compensated by increasing glucose uptake under acetic acid stress.

### Changes in lipid profiles in CNTR, DAG*^EN^*, DAG*^EN^*_Dgkα, and PM OF DAG*^EN^*_Dgkα in response to 9 g/l acetic acid

**Fig 5:**
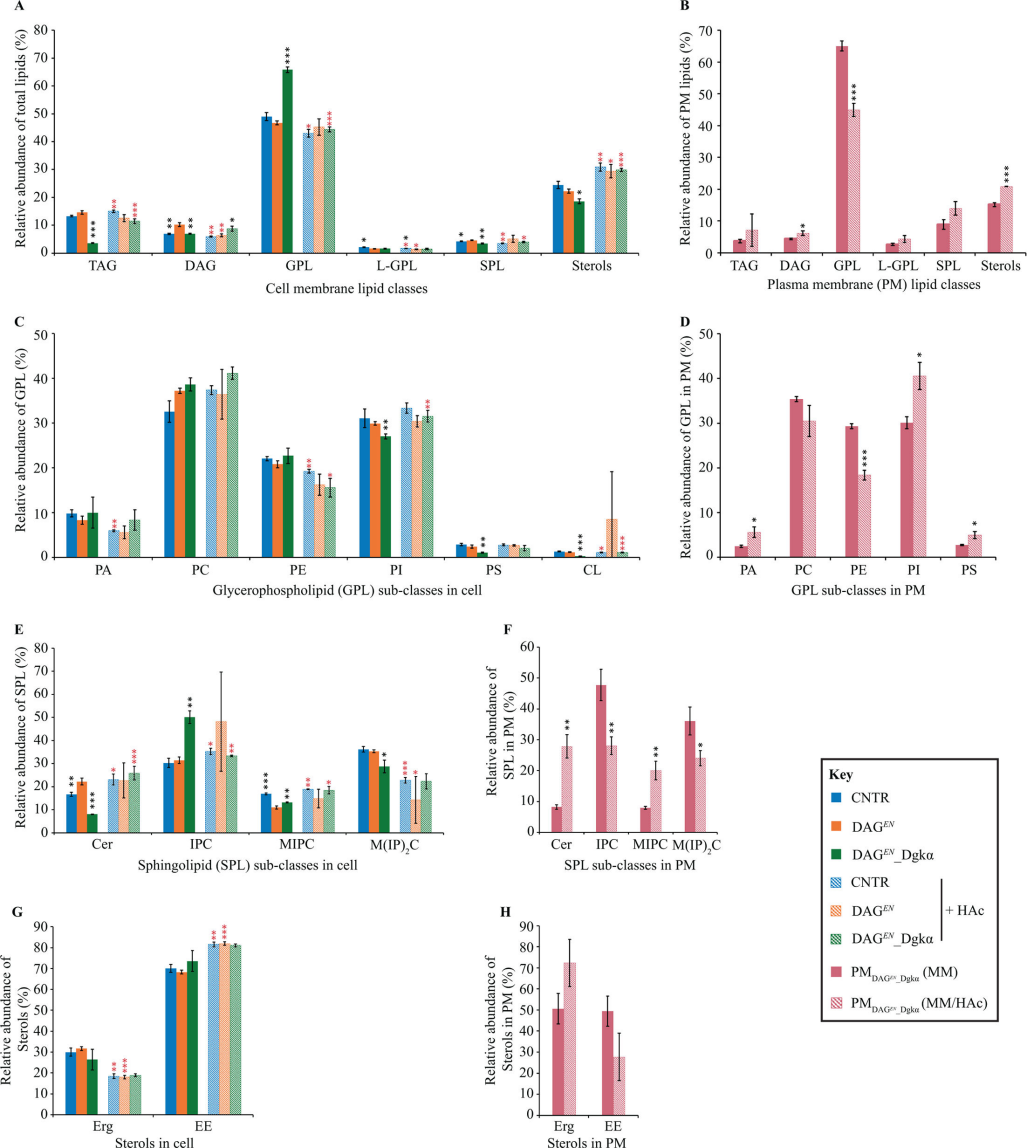
Lipid profiles in CNTR, DAG*^EN^*, and DAG*^EN^*_Dgkα cells show an adaptable lipid composition. (**A**) Relative abundance of lipid classes in total cell membranes of indicated strains under either standard or acid stress conditions. (**B**) Relative abundance of lipid classes in the PM of DAG*^EN^*_Dgkα cells under the indicated growth conditions. (**C, D**) Relative abundance of GPL sub-classes in total cell membranes from all strains (**C**) or in the PM of DAG*^EN^*_Dgkα cells (**D**) under the indicated growth conditions. (**E, F**) Relative abundance of SPL sub-classes in total cell membranes from all strains (**E**) or in the PM of DAG*^EN^*_Dgkα cells (**F**) under the indicated growth conditions. (**G, H**) Relative abundance of sterol sub-classes in total cell membranes from all strains (**G**) or in the PM of DAG*^EN^*_Dgkα cells (**H**) under the indicated growth conditions. Data represent the means ± standard deviation (*n* = 3). Student’s *t-test* was applied for intergroup comparison. *P < 0.05, **P < 0.01, and ***P < 0.001. Stars in black color represent significant differences between DAG*^EN^* and CNTR/ DAG*^EN^*_Dgkα under each growth condition. Stars in red color represent significant differences for each strain under two different growth conditions. TAG, triacylglycerol; DAG, diacylglycerol; GPL, glycerophospholipid; L-GPL, lyso-glycerophospholipid; SPL, sphingolipid; PA, phosphatidic acid; PC, phosphatidylcholine; PE, phosphatidylethanolamine; PI, phosphatidylinositol; PS, phosphatidylserine; CL, cardiolipin; Cer, ceremide; IPC, inositol phosphorylceramide; MIPC, mannosyl-inositol phosphorylceramide; M(IP)_2_C, mannosyl-di-(inositol phosphoryl) ceramide; Erg, ergosterol; EE, ergosterol ester.

Next, exponential phase cultures of CNTR, DAG*^EN^*, and DAG*^EN^*_Dgkα were collected from bioreactors and subjected to mass spectrometry (MS)-based shotgun lipidomics to determine total cell membrane lipid content under either standard or acetic acid conditions (Fig. 5). The same lipidomics analysis was performed also on PM-enriched fractions obtained from either standard or acetic acid DAG*^EN^*_Dgkα cultivations (Fig. 5). Expectedly, the PM was the major component in PM-enriched fractions, with PM H^+^-ATPase Pma1 (32) appearing as the main band on western blots (Fig. S2A). Combined with a vanadate-sensitive ATPase assay, the amount of PM in PM-enriched fractions was estimated at 75%.

Lipidomics confirmed the higher relative abundance of DAG in DAG*^EN^* compared to CNTR (14). In addition, it revealed a significantly lower DAG content in DAG*^EN^*_Dgkα than in DAG*^EN^*, which was comparable to that in CNTR cells (Fig. 5A). When 9 g/L acetic acid was added to the medium, the relative abundance of DAG decreased in both CNTR and DAG*^EN^*, but not in DAG*^EN^*_Dgkα (Fig. 5A). These data suggested that the DAG level could be restored in DAG*^EN^* cells upon expression of Dgkα. As GPL elongated fatty acyl chains were more abundant in DAG*^EN^*_Dgkα than CNTR cells under both growth conditions (Fig. S2B), restoring DAG abundance in DAG*^EN^* cells had no bearing on GPL acyl chain length.

Lipid profiles revealed that GPL was the main type of membrane lipid in both total cell membranes of all strains and the PM of DAG*^EN^*_Dgkα under stressful or standard conditions (Fig. 5A and B). Notably, in the absence of acetic acid, GPL accounted for 70% of PM total cell membrane lipids in DAG*^EN^*_Dgkα and 50% in CNTR and DAG*^EN^* (Fig. 5A). Under the same conditions, DAG*^EN^*_Dgkα displayed a significant less value in TAG (3%) compared with CNTR (11%) and DAG*^EN^* (12%) (Fig. 5A). Moreover, compared with CNTR and DAG*^EN^*, DAG*^EN^*_Dgkα exhibited the least SPL and sterols under standard conditions (Fig. 5A), further indicating how acyl-CoA was diverted toward producing membrane GPL (Fig. 1). The lipidomic data confirmed that *DGKα* expression in DAG*^EN^* directed DAG metabolism to GPL biosynthesis (Fig. 1).

In the presence of 9 g/L acetic acid, all membrane lipids except DAG exhibited a similar relative abundance among CNTR, DAG*^EN^*, and DAG*^EN^*_Dgkα (Fig. 5A). However, a significant drop in GPL as well as a rise in TAG, DAG, SPL, and sterols were observed in DAG*^EN^*_Dgkα cells. Similarly, CNTR exhibited more TAG and sterols, but fewer GPL, lyso-glycerophospolipids (L-GPL), and SPL. In DAG*^EN^*, only sterols showed an increase while DAG and L-GPL decreased when compared to non-stressed cells (Fig. 5A). The data imply that the membrane lipids were remodeled initially to the ratio usually observed in stressed yeast. The active membrane lipid adaptation in DAG*^EN^*_Dgkα suggested that the initial stress in DAG*^EN^* cells might be deriving from the enrichment in DAG, which could be relieved by expressing *DGKα*.

Further analysis of the PM obtained from DAG*^EN^*_Dgkα aimed to determine how lipid composition changed in response to acetic acid at the PM level. Similar to total cell membranes, GPL were the main lipids in the PM of DAG*^EN^*_Dgkα cells cultivated under both growth conditions although their relative abundance decreased in response to acetic acid (Fig. 5A and B). Given that SPL are enriched in the PM compared to other organelles (33), it was not unexpected that their relative abundance was 10% in the PM and only 5% in total cell membranes under any of the conditions tested (Fig. 5A and B). The rise in SPL in total cell membranes, but not in PM, of acid-stressed DAG*^EN^*_Dgkα cells indicated that either the amount of SPL in the PM was saturated or the efficiency of transferring SPL to the PM was low. The amount of DAG and sterols was increased in the PM of DAG*^EN^*_Dgkα cells cultivated in the acid-containing medium as opposed to standard conditions (Fig. 5B).

#### Glycerophospholipids

Phosphatidylcholine (PC), phosphatidylinositol (PI), and phosphatidylethanolamine (PE) were major components of total cell membranes under both growth conditions (Fig. 5C). The relative abundance of PI was lower in DAG*^EN^*_Dgkα than CNTR and DAG*^EN^* under standard conditions. This might be related to the consumption of PI for synthesizing complex SPL (Fig. 1), as suggested by a higher level of inositolphosphorylceramide (IPC) in DAG*^EN^*_Dgkα but not in CNTR and DAG*^EN^* (Fig. 5E). By contrast, DAG*^EN^*_Dgkα contained less phosphatidylserine (PS) and cardiolipin (Fig. 5C). The relative abundance of GPL sub-classes was similar in all cell types under acetic acid stress. PA, PE, and cardiolipin were less abundant in CNTR cells, whereas DAG*^EN^*_Dgkα cells contained more PI and cardiolipin, but less PE. No obvious change was observed for GPL sub-classes in DAG-enriched DAG*^EN^* cells (Fig. 5C).

Cardiolipin was not included in the quantification of GPL sub-classes in the PM of DAG*^EN^*_Dgkα cells because it is a mitochondrial membrane lipid (34). As shown in Fig. 5D, PC, PE, and PI were major GPL in the PM under both growth conditions. In response to acetic acid, the relative abundance of PI increased from 30% to 40% and became the most abundant GPL in the PM, along with PA and PS. By contrast, the amount of PE decreased from 30% to less than 20% upon the addition of acetic acid (Fig. 5D).

#### Sphingolipids

The distribution of SPL sub-classes in total cell membranes of CNTR, DAG*^EN^*, and DAG*^EN^*_Dgkα, as well as in the PM of DAG*^EN^*_Dgkα is shown in Fig. 5E and F. Under standard conditions, DAG*^EN^*_Dgkα and CNTR presented less ceramide than DAG*^EN^* in total cell membranes (Fig. 5E). Both CNTR and DAG*^EN^*_Dgkα contained more mannosyl-inositol phosphorylceramide (MIPC) than DAG*^EN^* (Fig. 5E), whereas DAG*^EN^*_Dgkα had less mannosyl-di-inositolphosphorylceramide (M(IP)_2_C) than CNTR and DAG*^EN^* (Fig. 5E). SPL sub-classes in all three strains were adjusted to a similar level in response to 9 g/L acetic acid (Fig. 5E). The amount of M(IP)_2_C was decreased in CNTR and DAG*^EN^* cells, whereas ceramide, IPC, and MIPC were increased in DAG*^EN^*. DAG*^EN^*_Dgkα cells exhibited more ceramide and MIPC, but less IPC (Fig. 5E). Overall, IPC and M(IP)_2_C were the main components of SPL in the PM of DAG*^EN^*_Dgkα when acetic acid was absent. In the presence of acetic acid, ceramide and MIPC became more abundant in the PM, at the expense of IPC and M(IP)_2_C (Fig. 5F).

#### Sterols

Ergosterol ester (EE) was the main sterol in total cell membranes of CNTR, DAG*^EN^*, and DAG*^EN^*_Dgkα cells regardless of growth condition (Fig. 5G). Overall, the relative abundance of ergosterol and EE was similar in all three yeast strains, although an increase in EE and decrease in ergosterol was observed in CNTR and DAG*^EN^* cells, but not in DAG*^EN^*_Dgkα cells, cultivated in acetic acid-containing medium (Fig. 5G). Under standard conditions, the relative abundance of ergosterol and EE in the PM of DAG*^EN^*_Dgkα cells was similar; however, ergosterol increased slightly to around 70% in response to acetic acid (Fig. 5F).

Taken together, lipidomics revealed that the relative abundance of all membrane lipid species in CNTR, DAG*^EN^*, and DAG*^EN^*_Dgkα changed to similar levels in response to 9 g/L acetic acid. However, the change in membrane lipid composition was more pronounced in cells expressing *DGKα*. Furthermore, it cannot be excluded that the active membrane remodeling might play a role in improving the acetate excretion in acetic acid-stressed DAG*^EN^*_Dgkα cells.

## DISCUSSION

The PM is the outermost physical barrier of the cell (7), membrane engineering aims to decrease PM permeability to undissociated acetic acid by changing its lipid compositions (14, 35). Our previous studies have shown that it is possible to engineer the PM to achieve a targeted composition or specific lipid features although the overall effect on the membrane physicochemical properties is not predictable. For example, the *S. cerevisiae* DAG*^EN^* strain attained longer fatty acid chains on GPL, which likely enhanced membrane thickness according to molecular dynamic simulations but failed to slow down acetic acid net uptake. This unexpected result could be due to an abnormally high accumulation of DAG, which might disrupt the PM lamellar structure and compromise its properties (14).

Therefore, following an iterative approach, we applied strain engineering to restore the DAG level in DAG*^EN^* cells, to understand how membrane lipid compositions and their physicochemical properties were linked to cell physiology. Lipidomics showed that the relative abundance of DAG was successfully recovered upon expression of Dgkα (Fig. 5A), without affecting GPL acyl chain length (Fig. S2B). DAG*^EN^*_Dgkα cells grew better than DAG*^EN^* in the presence of 13 g/L acetic acid (Fig. 2). Crucially, this was not due to decreased membrane permeability to acetic acid (Fig. 3A), but rather related to an increased capacity to maintain low intracellular acetic acid, regardless of uptake (Fig. 3B).

The lower acetic acid content in DAG*^EN^*_Dgkα could be the result of increased acetate efflux across the PM. Intracellular pH drops after acetic acid dissociates inside cells. As a response, the H^+^-ATPase Pma1 in the PM pumps out protons to maintain a stable intracellular pH, along with the export of acetate by PM transporters Top2, Top3, and Aqr1 (36). These transporters, along with Pma1, play a key role in dictating yeast acetic acid resistance (37–39). The PM in DAG*^EN^*_Dgkα likely favors the active excretion of protons and acetate following changes in lipid composition caused by the engineered GPL biosynthesis pathway. In addition, both proton and acetate efflux are highly energy demanding, consuming more than 20% of total ATP produced by glucose-grown cells (40). Consistently, several signs of energy depletion were observed in DAG*^EN^*_Dgkα cultivated in standard conditions, including lower specific growth rate (Fig. 2; Fig. S1), lower biomass yield (Table 1), and a high percentage of metabolically inactive cells (Fig. 4B). Under acetic acid stress, DAG*^EN^*_Dgkα cells behaved similarly to CNTR and DAG*^EN^*. An increased glucose uptake rate was observed in DAG*^EN^*_Dgkα in response to 9 g/L acetic acid, indicating that cells boost their energy supply to maintain their activity and resistance to acetic acid. Molecular dynamic simulations and lipidomics indicated that *Z. bailii* increases the content of SPL in the PM during acetic acid stress (12, 13). Here, an increase in SPL was observed in DAG*^EN^*_Dgkα cell membranes (Fig. 5A), but not specifically in the PM (Fig. 5B), pointing to defects in the transport of SPL to the PM in DAG*^EN^*_Dgkα cells. In addition, lipidomics showed that all membrane lipids were adjusted to a similar level in all three strains under acetic acid conditions (Fig. 5). The ability to adapt was more pronounced in CNTR and DAG*^EN^*_Dgkα, whereas DAG*^EN^* changed only their DAG and ergosterol content in response to acetic acid. These results imply that the ability to vary membrane lipid ratios in DAG*^EN^* cells was improved upon expression of Dgkα, likely allowing these cells to withstand even harsher (13 g/L) acetic acid stress.

### Conclusion

In the present study, we successfully engineered a yeast strain that could grow under high acetic acid stress by regulating its diacylglycerol metabolism. We compared how the plasma membrane and total cell membranes responded to acetic acid by adjusting their lipid content. By combining physiological and lipidomics analyses in DAG*^EN^*_Dgkα cells cultivated in the absence or presence of acetic acid, we found that the capacity of the membrane to adapt lipid composition together with sufficient energy supply influenced membrane properties in response to stress. We suggest that potentiating the intracellular energy system or enhancing lipid transport to destination membranes should be taken into account when designing membrane engineering strategies.

## MATERIALS AND METHODS

### Strains and growth conditions

The *S. cerevisiae* strains used in this study are listed in Table 2. Yeast cells were grown at 30°C on minimal medium (41) containing 20 g/L glucose as a carbon source. To maintain the pH at 5.0 when cultivating yeast cells in either Erlenmeyer flasks or 96-well plates, 50 mM potassium hydrogen phthalate was added to the medium. When required, acetic acid (pH 5.0) was added to a final concentration of 9 g/L or 13 g/L. Yeast transformants were selected on YPD plates (10 g/L yeast extract, 20 g/L peptone, 20 g/L glucose, and 20 g/L agar) containing 200 mg/L G418 sulfate (Thermo Scientific).

**TABLE 2:** List of yeast strains used in this study

Strain	Characteristics	Reference
CNTR	CEN.PK 113_5D with YTK; *MATα*, *SUC2*, *MAL2-8^c^*, *ura3-52::URA3*	(14)
DAG*^EN^*	CNTR with *FAE1*_*GPAT5*; *MATα*, *SUC2*, *MAL2-8^c^*, *ura3-52::URA3*	(14)
DAG*^EN^*_Dgkα	DAG*^EN^* with P*_ALD6_*_*DGKα*; *MATα*, *SUC2*, *MAL2-8^c^*, *ura3-52::URA3*	This study

### Construction of *S. cerevisiae* DAG*^EN^*_Dgkα

The fragment encoding full-length *DGKα* was synthesized by TWIST Bioscience and integrated into a vector harboring the *ALD6* promoter along with homology arms for the *HO locus* using the MoClo modular cloning system plasmid kit for *S. cerevisiae* (42) to obtain plasmid P*_ALD6_-DGKα*. Gene integration was carried out using the LiAc/salmon sperm carrier DNA/polyethylene glycol method (43) in combination with CRISPR/Cas9 for improved integration efficiency (44). YN2_1_Cas9 was the backbone Cas9 plasmid used in this study (45). Yeast transformants were verified by colony PCR using primers [F: GCTCTACGAGCCTGTTGGAG (5′ to 3′)] and [R: CCGTGCCTGCGATGAGATAC (5′ to 3′)]. The Cas9 plasmid was cured by re-streaking positive clones twice on antibiotic-free YPD plates.

### Cultivation of *S. cerevisiae* strains

*S. cerevisiae* strains were inoculated in 100 mL Erlenmeyer flasks containing 10 mL minimal medium (pH 5.0) and incubated overnight at 30°C and 200 rpm.

For cultivation in a growth profiler (GP960 REV2; EnzyScreen B.V.), cells from an overnight pre-culture were transferred to a clear-bottom 96-well plate (CR1496e; System Duetz) in 200 µL minimal medium containing 0 g/L, 9 g/L, or 13 g/L acetic acid and at a starting OD_600_ of 0.05. The plate was covered with an aerobic cover (CR1296a; EnzyScreen B.V.) and incubated for 120 h at 30°C and 250 rpm. Green light scattering was measured every 30 min and the values were converted to OD_600_ using a standard curve previously plotted using *S. cerevisiae* CEN.PK_113.7D grown in minimal medium (14). Growth medium was used as the blank.

Aerobic batch cultivations were conducted in 2.5 L bioreactors (Labfors4; INFORS-HT) with an initial working volume of 2 L minimal medium containing either 0 g/L or 9 g/L acetic acid. The initial yeast cell density was around OD_600_ = 0.05. Temperature was maintained at 30°C and pH at 5.0 by automatically adding either 2 M KOH or 1 M HCl. The bioreactors were sparged with an initial constant flow of air at 2 VVM, which was then switched to 1 VVM after collecting 1 L of mid-exponential phase cell cultures for PM fractionation. The initial stirring speed was set to 300 rpm to ensure a dissolved oxygen tension of at least 30% air saturation. Oxygen and CO_2_ concentrations were monitored with a DASGIP Off-Gas Analyzer GA4, with 0.2 mL of silicone antifoam (Sigma-Aldrich) added to prevent excessive foaming. At regular intervals, 12 mL culture samples were taken for subsequent determination of biomass and extracellular metabolites, including glucose, ethanol, glycerol, acetate, and pyruvate.

### Dry cell weight

Dry cell weight was determined as described before (12) with minor modifications. Duplicate 5 mL cell culture samples were filtered through dry, pre-weighted 0.45 µm PES membrane filters (Sartorius Biolab). The biomass retained on the filter was washed twice with 20 mL deionized water, dried in a microwave oven at 120 W for 15 min, and then stored in a silica-gel desiccator for one day before weighing.

### Analysis of substrate and extracellular metabolites

Briefly, 1 mL samples collected from the bioreactors were immediately filtered through 0.2 µm nylon syringe filters (VWR International) and stored at −20°C until analysis or loaded on a high-performance liquid chromatographer (Jasco UV-RI HPLC detector) equipped with an Rezex ROA-Organic Acid H^+^ (8%) column (Phenomenex) maintained at 60°C. Samples were eluted with 5 mM H_2_SO_4_ at a flow rate of 0.6 L/min. Glucose, ethanol, glycerol, pyruvate, and acetic acid were quantified as described previously (12).

### Calculation of physiological parameters

All data are presented as the mean ± standard deviation of biological triplicates. The μ_Max_, Y_i/S_ from the total consumed substrate, and glucose uptake rate (q_Glu_) were calculated for exponentially growing cells as described before (12). Specifically, μ_Max_ was determined as the slope of the linear curve obtained when plotting the biomass-to-glucose carbon molar ratio. Similarly, Y_i/S_ was determined as the slope of the linear curve when plotting each extracellular metabolite-to-glucose carbon molar ratio. Finally, q_Glu_ was calculated according to the equation $q_{Glu}=\mu_{Max}\;/\;Y_{X/S}.$

### Acetic acid uptake and kinetic measurements

Acetic acid diffusion kinetics were determined as described previously (14). For the kinetics experiment, a final 7.4–51.8 kBq (0.148–1.036 Bq/µL) [1-^14^C] acetic acid (NEC084H001MC; PerkinElmer) was mixed with 0.2–1.4 mM non-radiolabeled acetic acid, resulting in a total of 0.27–1.9 mM acetic acid, with a specific activity of 39,300 DPM/nmol. Each assay was initiated by incubating 10 µL of cells stored on ice with 30 µL of 50 mM potassium hydrogen phthalate buffer at pH 5.0, in a 30°C water bath for 4 min. Acetic acid diffusion was measured after the addition of 10 µL acetic acid mixture and incubation at 30°C for 30 s. The reaction was stopped by adding 10 mL of ice-cold 2 mM non-radiolabeled acetic acid stop solution. The cells were swiftly filtered and washed with another 10 mL ice-cold 2 mM non-radiolabeled acetic acid. The filters (Ø 25 mm; Whatman) were then placed in vials with 12 mL Emulsifier-Safe scintillation liquid (PerkinElmer) and shaken thoroughly.

Acetic acid diffusion was measured by mixing a final 1 µCi (20 nCi/µL) [^14^C] acetic acid with 2 mM unlabeled acetic acid, which had a specific activity of 16,600 DPM/nmol. Each assay was initiated by incubating 60 µL of cells stored on ice with 180 µL of 50 mM potassium hydrogen phthalate buffer at pH 5.0 in a 30°C water bath for 4 min. Acetic acid diffusion was measured after adding 60 µL acetic acid mixture. Samples of 50 µL were withdrawn from the diffusion assay after 0 s, 15 s, 40 s, 70 s, 5 min, and 10 min, and added to 10 mL of an ice-cold stop solution containing 2.4 mM non-radiolabeled acetic acid. The stop solution containing the cells was rapidly filtered through GF/C filters (Ø 25 mm, Whatman). The filters were then washed with 10 mL stop solution and placed in vials with 12 mL Emulsifier-Safe scintillation liquid and shaken thoroughly.

The amount of intracellular acetic acid was determined by measuring the radioactive decay of [^14^C] acetic acid using a liquid scintillation counter (Wallac Guardian 1414; PerkinElmer) as described before (25). Background radiation levels were determined in cultures, scintillation liquid, and filters. None of the background controls showed significant amounts of radioactivity, and the average background was subtracted from the samples. The measured radioactive decay was within the linear concentration range and no quenching of the sample matrix was observed.

### Plasma membrane isolation

The yeast PM was obtained following a standard protocol (46) with some adjustments. Samples (1 L of cell culture) collected from bioreactors were centrifuged (4000 rpm, 4°C, 10 min) and washed twice with ice-cold deionized water. Cell pellets were resuspended in 5 mL buffer A (25 mM imidazole-HCl, 0.4 M sucrose, pH 7,0) containing a protease inhibitor cocktail (Halt 100×; Thermo Scientific). The cells were thoroughly lysed with 1.5 volumes of glass beads until more than 80% of the cells were broken, as assessed by microscopy. Finally, 500 µL of cell lysates was snap-frozen and stored at −80°C for subsequent whole-cell lysate lipidomics.

For PM isolation, the lysed cells were centrifuged at 600 × *g* and 4°C for 20 min, after which the supernatant was collected and centrifuged at 22,000 × *g* and 4°C for 30 min. The pellet was resuspended in 2 mL buffer A and 1 mL of the suspension was gently placed on top of a 12 mL discontinuous sucrose gradient (consisting of three layers of 2.2 M, 1.65 M, and 1.1 M sucrose in 25 mM imidazole-HCl, pH 7.0). Gradient centrifugation was conducted in an Optima LE-80K preparative ultracentrifuge (Beckman Coulter) at 30,000 rpm for 4 h. PM-enriched fractions were collected from the interface between the 2.25 M and 1.65 M sucrose layers, resuspended in 4 volumes of 25 mM imidazole-HCl (pH 7.0), and pelleted again by centrifugation at 18,000 rpm and 4°C for 40 min. The pellet was resuspended in 500 µL ice-cold PBS buffer (pH 7.0) and stored at −80°C for subsequent PM lipidomics.

### Western blotting

Yeast cell lysates, total cell membrane samples, and PM-enriched fractions were denatured in 4× SDS loading buffer (0.2 M Tris-HCl, 0.4 M DTT, 8.0% SDS, 6 mM bromophenol blue, 4.3 M glycerol, and 2% β-mercaptoethanol). Equal amounts of protein, whose concentration was determined by conducting Bradford assay, were loaded on a gel and blots were probed using antibodies against Pma1 (1:1,000 dilution; Abcam), Vma2 (1:2,000 dilution; Abcam), Dpm1 (1:250 dilution; Abcam), and Cox4 (1:1,000 dilution; Abcam). All reaction steps followed the Pierce Fast Western Blot Kit protocol (Thermo Scientific). Blots were scanned using ChemiDoc MP Imaging (Bio-Rad laboratories).

### Lipidomics

MS-based lipid analysis was performed by Lipotype GmbH (Dresden, Germany) as described previously (47, 48). Lipids were extracted using a two-step chloroform/methanol procedure (47). Samples were spiked with an internal lipid standard mixture containing: CDP-DAG 17:0/18:1, cardiolipin 14:0/14:0/14:0/14:0, ceramide 18:1;2/17:0, DAG 17:0/17:0, lyso-phosphatidate (LPA) 17:0, lyso-phosphatidyl-choline (LPC) 12:0, lyso-phosphatidylethanolamine (LPE) 17:1, lyso-phosphatidylinositol (LPI) 17:1, lyso-phosphatidylserine (LPS) 17:1, PA 17:0/14:1, PC 17:0/14:1, PE 17:0/14:1, phosphatidylglycerol (PG) 17:0/14:1, PI 17:0/14:1, PS 17:0/14:1, EE 13:0, TAG 17:0/17:0/17:0, stigmastatrienol, IPC 44:0;2, MIPC 44:0;2, and M(IP)_2_C 44:0;2. After extraction, the organic phase was transferred to an infusion plate and dried in a speed vacuum concentrator. First, the dry extract was resuspended in 7.5 mM ammonium acetate in chloroform/methanol/propanol (1:2:4, vol:vol:vol); then, the dry extract was resuspended in 33% ethanol solution of methylamine in chloroform/methanol (0.003:5:1, vol:vol:vol). All liquid handling steps were performed using a Hamilton Robotics STARlet robotic platform with the Anti Droplet Control feature for organic solvent pipetting.

### MS data acquisition in lipidomic analysis and data analysis

Samples were analyzed by direct infusion on a QExactive mass spectrometer (Thermo Scientific) equipped with a TriVersa NanoMate ion source (Advion Biosciences). Samples were analyzed in both positive and negative ion modes, with a resolution of 280,000 at *m/z* = 200 for MS and 17,500 at *m/z* = 200 for MS/MS, in a single acquisition. MS/MS was triggered by an inclusion list encompassing corresponding MS mass ranges scanned in 1 Da increments (49). Both MS and MS/MS data were combined to monitor EE, DAG, and TAG ions as ammonium adducts; PC as an acetate adduct; and cardiolipin, PA, PE, PG, PI, and PS as deprotonated anions. MS alone was used to monitor CDP-DAG, LPA, LPE, LPI, LPS, IPC, MIPC, M(IP)_2_C as deprotonated anions; ceramide and LPC as acetate adducts and ergosterol as protonated ion of an acetylated derivative (50).

Data were analyzed with in-house developed lipid identification software based on LipidXplorer (51, 52). Data post-processing and normalization were performed using an in-house developed data management system. Only lipid identifications with a signal-to-noise ratio >5, and a signal intensity fivefold higher than in corresponding blank samples were considered for further analysis.

### Determination of yeast cell metabolic activity

Intracellular metabolic activity was measured using the Live/Dead Yeast Viability Kit (Thermo Scientific). Fluorescent dyes Calcofluor White (1 µL) and FUN-1 (2 µL) were added to 5 mL of mid-exponential phase cell cultures and incubated at 30°C for 30 min. Images were captured at room temperature with a confocal laser scanning microscope (Nikon Ti-E A1+) using a 60 × 1.40 objective. FUN-1 was excited at 488 nm and emitted at 530 nm (green) and 620 nm (red). The excitation and emission wavelengths for Calcofluor White were 385 nm and 475 nm. Image analysis was performed using ImageJ, and figures were prepared using Adobe Illustrator software.

Live, dead, and impaired cells were quantified using 300 randomly selected cells from three independent cultures. Cells with red CIVS were read as “live,” cells with red cytosolic spots were read as “impaired,” and cells emitting only green fluorescence were read as “dead.” Standard deviations were calculated using Microsoft Excel. Significance was determined using a two-tailed Student’s *t*-test, with *P < 0.05, **P < 0.01, and ***P < 0.001.

### Spot assay

Exponentially growing cells in glucose were harvested by centrifugation and diluted to an OD_600_ of 1.0 in water. Serial dilutions were made (10^−1^, 10^−2^, 10^−3^, 10^−4^, and 10^−5^) and spotted on YP plates containing glucose (0.02% wt/vol) or glycerol (0.02%vol/vol) as carbon source. The plates were incubated at 30°C for 4 days.

## Data Availability

The raw lipidomics data obtained through Lipotype GmbH are included in this article as Table S1.

## SUPPLEMENTAL MATERIAL

Supplemental figures aem.01212-24-s0001.pdf Figures S1 and S2.

Table S1 aem.01212-24-s0002.xlsx Raw lipidomics data.

ASM does not own the copyrights to Supplemental Material that may be linked to, or accessed through, an article. The authors have granted ASM a non-exclusive, world-wide license to publish the Supplemental Material files. Please contact the corresponding author directly for reuse.
