# Supplementary material for: Exploring the interplay between yeast cell membrane lipid adaptation and physiological response to acetic acid stress
Source: Appl Environ Microbiol. 2024 Nov 13;90(12):e01212-24. doi: 10.1128/aem.01212-24 (PMC11654797; doi:10.1128/aem.01212-24)
Supplement: Supplemental figures — Figures S1 and S2. [file aem.01212-24-s0001.pdf]

Supplemental Information for:

Exploring the interplay between yeast cell membrane lipid adaptation and physiological response to acetic acid stress

Fei Wu [1], Maurizio Bettiga [1, 2] Lisbeth Olsson [1,] *,

1 Department of Life Sciences, Division of Industrial Biotechnology, Chalmers University of Technology, 412 96 Gothenburg, Sweden

2 Italbiotec Srl Benefit Corporation, Innovation Unit, 20126 Milan, Italy

* Corresponding author: lisbeth.olsson@chalmers.se

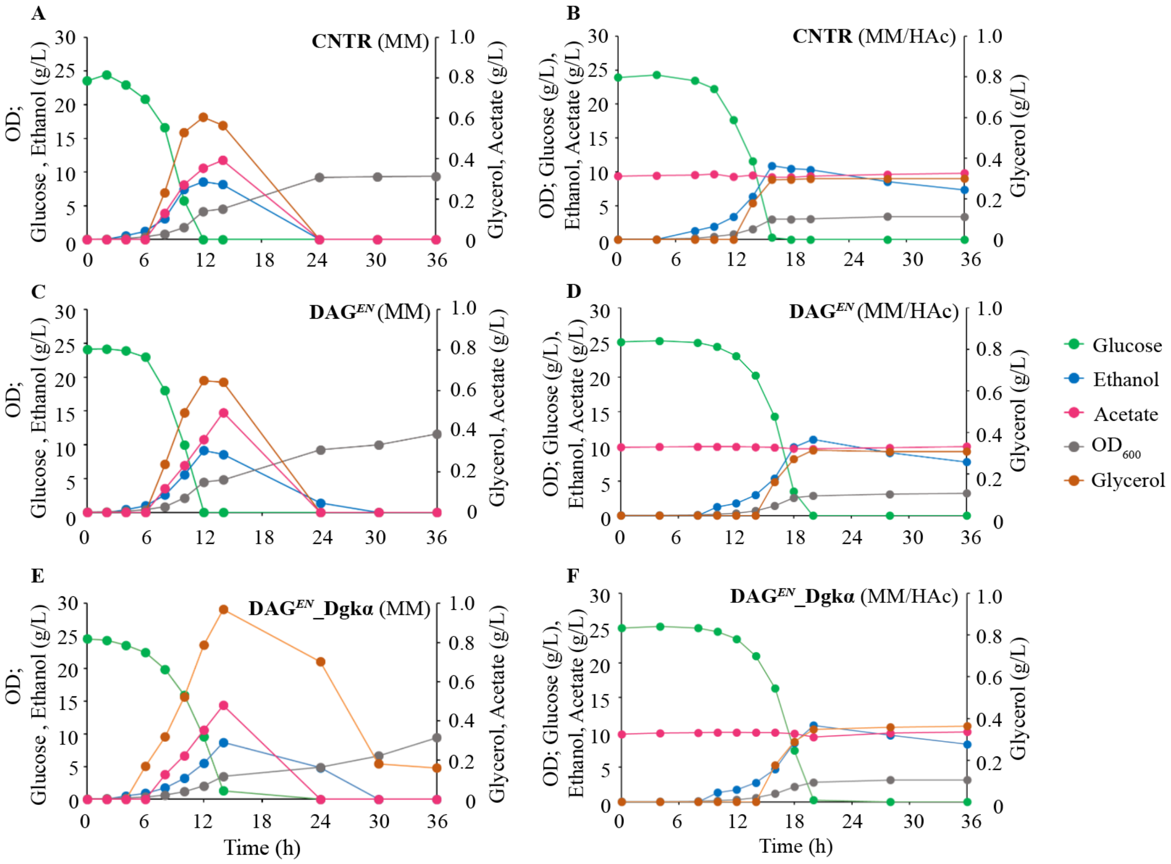

Figure S1. Fermentation profiles of strain CNTR, DAG^{EN}, DAG^{EN}_Dgkα cultured with 9 g/L and without acetic acid. Cell density (optical density at 600 nm) and extracellular concentrations of glucose, ethanol, glycerol, and acetate during the batch cultivation of (A) CNTR in mineral medium (MM); (B) CNTR in mineral medium with 9 g/L acetic acid (MM/HAc); (C) DAG^{EN} in mineral medium; (D) DAG^{EN} in the mineral medium with 9 g/L acetic acid; (E) DAG^{EN}_Dgkα in mineral medium; (F) DAG^{EN}_Dgkα in the mineral medium with 9 g/L acetic acid. A representative profile from three independent cultures is shown for each strain under each growth condition.

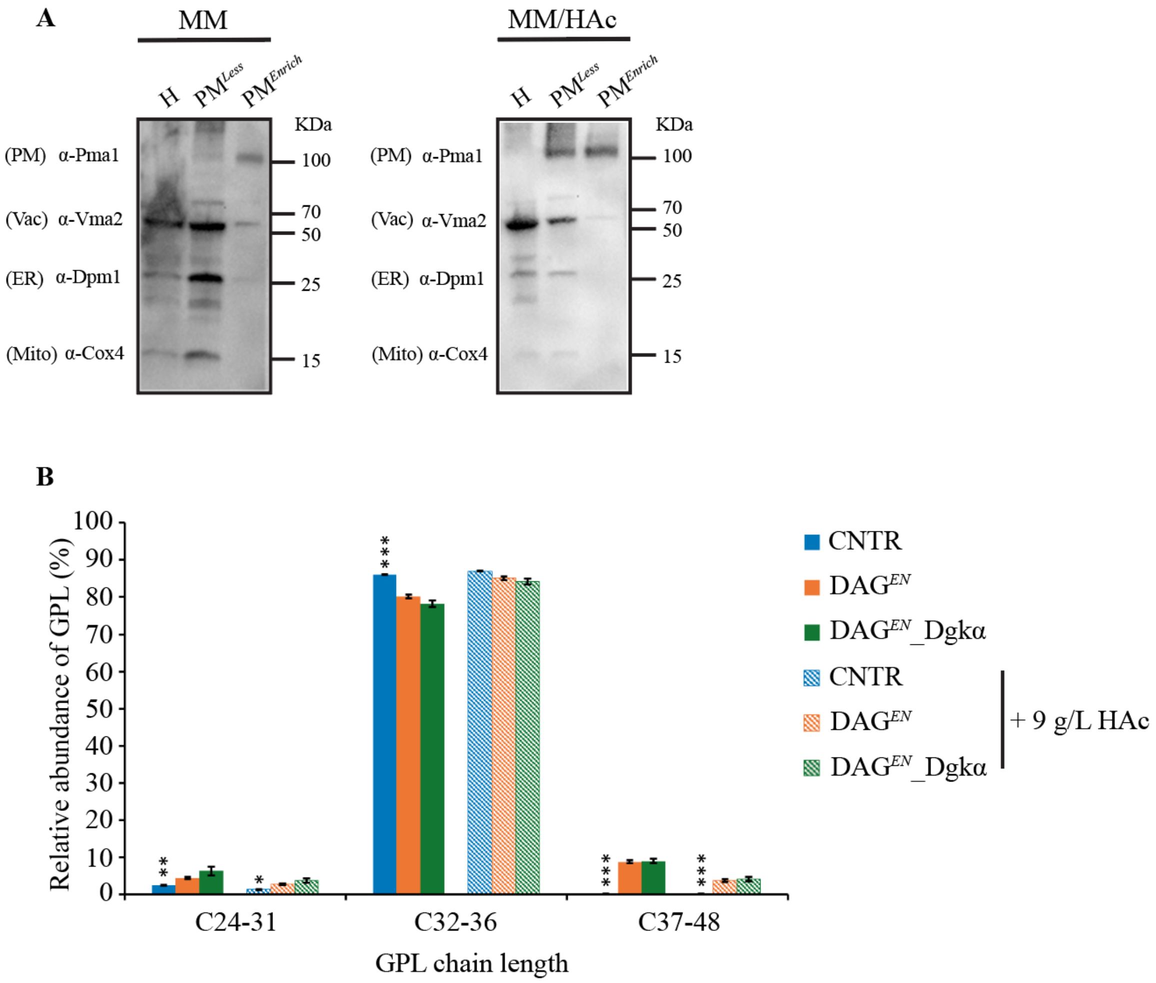

Figure S2. Western blot of membrane fractions in the plasma membrane samples sent for lipidomics and quantification of Glycerophospholipid acyl chain length in whole cell lysate. (A) The blot was decorated with α-Pma1, α-Vma2, α-Dpm1 and α-Cox4 antibodies. Cells were grown till the mid-exponential phase in bioreactors on mineral medium (MM) or mineral medium with 9 g/L acetic acid (MM/HAc). A representative blot from three independent cultures is shown. H, homogenise; PM^{Less}: plasma membrane-less fraction, which are membrane samples collected from 1.65M and 1.1M sucrose crosslayer; PM^{Enrich}: plasma membrane-enrich fraction, which are membrane samples collected from 2.2 M and 1.65 M sucrose crosslayer. PM, plasma membrane; Vac, vacuolar; ER, endoplasmic reticulum; Mito, mitochondrion. (B) The combined chain length of the two acyl chains of glycerophospholipids obtained from total lipid analysis of CNTR, DAG^{EN} and DAG^{EN}_Dgkα under either standard or acid stress conditions. Data are means from three independent experiments and error bars represent standard deviation. Two tail student *t*-test has been performed, “*” = $p < 0.05$, “**” = $p < 0.01$ and “***” = $p < 0.001$. GPL, glycerophospholipid.
